# Supplementary material for: Generative and discriminative training of Boltzmann machine through quantum annealing
Source: Sci Rep. 2023 May 16;13:7889. doi: 10.1038/s41598-023-34652-4 (PMC10188519; doi:10.1038/s41598-023-34652-4)
Supplement: Supplementary file 1 — Supplementary Information. [file 41598_2023_34652_MOESM1_ESM.pdf]

Supplementary Information: Generative and discriminative training of Boltzmann machine through Quantum annealing

Siddhartha Srivastava*
Department of Mechanical Engineering
University of Michigan Ann Arbor
sidsriva@umich.edu

Veera Sundararaghavan
Department of Aerospace Engineering
University of Michigan Ann Arbor
veeras@umich.edu

Appendix

Appendix A Definition of statistical quantities

For completeness of notation, the definition of each statistical quantity is provided in context of the random variables used in this paper. Conditional quantities require following conditional probabilities:

$$p(\boldsymbol{h}|\boldsymbol{v};\boldsymbol{\theta},\beta) = \frac{p([\boldsymbol{v},\boldsymbol{h}];\boldsymbol{\theta},\beta)}{\sum_{\widetilde{\boldsymbol{h}}} p([\boldsymbol{v},\widetilde{\boldsymbol{h}}];\boldsymbol{\theta},\beta)}$$

$$p([\boldsymbol{v}^O,\boldsymbol{h}]|\boldsymbol{v}^I;\boldsymbol{\theta},\beta) = \frac{p([\boldsymbol{v}^I,\boldsymbol{v}^O,\boldsymbol{h}];\boldsymbol{\theta},\beta)}{\sum_{\widetilde{\boldsymbol{v}}^O,\widetilde{\boldsymbol{h}}} p([\boldsymbol{v}^I,\widetilde{\boldsymbol{v}}^O,\widetilde{\boldsymbol{h}}];\boldsymbol{\theta},\beta)}$$

A.1 Expectations

$$\mathbb{E}(E;\boldsymbol{\theta},\beta) = \sum_{\boldsymbol{S}} E(\boldsymbol{S};\boldsymbol{\theta})p(\boldsymbol{S};\boldsymbol{\theta},\beta)$$

$$\mathbb{E}\left(\frac{\partial E}{\partial\theta_i};\boldsymbol{\theta},\beta\right) = \sum_{\boldsymbol{S}} \frac{\partial E}{\partial\theta_i}(\boldsymbol{S};\boldsymbol{\theta})p(\boldsymbol{S};\boldsymbol{\theta},\beta)$$

$$\mathbb{E}\left(\frac{\partial E}{\partial\theta_i}\middle|\boldsymbol{v};\boldsymbol{\theta},\beta\right) = \sum_{\boldsymbol{h}} \frac{\partial E}{\partial\theta_i}([\boldsymbol{v},\boldsymbol{h}];\boldsymbol{\theta})p(\boldsymbol{h}|\boldsymbol{v};\boldsymbol{\theta},\beta)$$

$$\mathbb{E}\left(\frac{\partial E}{\partial\theta_i}\middle|\boldsymbol{v}^I;\boldsymbol{\theta},\beta\right) = \sum_{\boldsymbol{v}^O,\boldsymbol{h}} \frac{\partial E}{\partial\theta_i}([\boldsymbol{v}^I,\boldsymbol{v}^O,\boldsymbol{h}];\boldsymbol{\theta})p([\boldsymbol{v}^O,\boldsymbol{h}]|\boldsymbol{v}^I;\boldsymbol{\theta},\beta)$$

A.2 Covariances

The dependence on $\boldsymbol{\theta}$ and β is dropped for notational convenience.

$$\begin{aligned}
\mathrm{Cov}\left(\frac{\partial E}{\partial \theta_i}, \frac{\partial E}{\partial \theta_j}\right) &= \sum_{\boldsymbol{S}} \frac{\partial E}{\partial \theta_i}\frac{\partial E}{\partial \theta_j}(\boldsymbol{S})p(\boldsymbol{S}) - \mathbb{E}\left(\frac{\partial E}{\partial \theta_i}\right)\mathbb{E}\left(\frac{\partial E}{\partial \theta_j}\right)\\
\mathrm{Cov}\left(\frac{\partial E}{\partial \theta_i}, \frac{\partial E}{\partial \theta_j}\middle|\, \boldsymbol{v}\right) &= \sum_{\boldsymbol{h}} \frac{\partial E}{\partial \theta_i}\frac{\partial E}{\partial \theta_j}([\boldsymbol{v},\boldsymbol{h}])p(\boldsymbol{h}|\boldsymbol{v}) - \mathbb{E}\left(\frac{\partial E}{\partial \theta_i}\middle|\, \boldsymbol{v}\right)\mathbb{E}\left(\frac{\partial E}{\partial \theta_j}\middle|\, \boldsymbol{v}\right)\\
\mathrm{Cov}\left(\frac{\partial E}{\partial \theta_i}, \frac{\partial E}{\partial \theta_j}\middle|\, \boldsymbol{v}^I\right) &= \sum_{[\boldsymbol{v}^O,\boldsymbol{h}]} \frac{\partial E}{\partial \theta_i}\frac{\partial E}{\partial \theta_j}([\boldsymbol{v}^I,\boldsymbol{v}^O,\boldsymbol{h}])p([\boldsymbol{v}^O,\boldsymbol{h}]|\boldsymbol{v}^I) - \mathbb{E}\left(\frac{\partial E}{\partial \theta_i}\middle|\, \boldsymbol{v}^I\right)\mathbb{E}\left(\frac{\partial E}{\partial \theta_j}\middle|\, \boldsymbol{v}^I\right)
\end{aligned}$$

A.3 Variances

$$\begin{aligned}
\mathrm{Var}\,(E) &= \sum_{\boldsymbol{S}} E^2(\boldsymbol{S})p(\boldsymbol{S}) - \mathbb{E}^2\,(E)\\
\mathrm{Var}\,(E|\,\boldsymbol{v}) &= \sum_{\boldsymbol{h}} E^2([\boldsymbol{v},\boldsymbol{h}])p(\boldsymbol{h}|\boldsymbol{v}) - \mathbb{E}^2\,(E|\,\boldsymbol{v})\\
\mathrm{Var}\left(E|\,\boldsymbol{v}^I\right) &= \sum_{[\boldsymbol{v}^O,\boldsymbol{h}]} E^2([\boldsymbol{v}^I,\boldsymbol{v}^O,\boldsymbol{h}])p([\boldsymbol{v}^O,\boldsymbol{h}]|\boldsymbol{v}^I) - \mathbb{E}^2\left(E|\,\boldsymbol{v}^I\right)
\end{aligned}$$

Appendix B Estimation of gradients

B.1 Gradient of KL Divergence

The gradient of Log-likelihood for a single data is estimated as:

$$\begin{aligned}
\frac{\partial \ln p(\boldsymbol{v})}{\partial \theta_j} &= \frac{\partial}{\partial \theta_j}\left(\ln \sum_{\boldsymbol{h}} e^{-\beta E(\boldsymbol{v},\boldsymbol{h})}\right) - \frac{\partial}{\partial \theta_j}\left(\ln \sum_{\boldsymbol{v}',\boldsymbol{h}'} e^{-\beta E(\boldsymbol{v}',\boldsymbol{h}')}\right)\\
&= -\beta \sum_{\boldsymbol{h}} \frac{e^{-\beta E(\boldsymbol{v},\boldsymbol{h})}}{\sum_{\boldsymbol{h}'} e^{-\beta E(\boldsymbol{v},\boldsymbol{h}')}}\frac{\partial E(\boldsymbol{v},\boldsymbol{h})}{\partial \theta_j} + \beta \sum_{\boldsymbol{v}',\boldsymbol{h}'} \frac{e^{-\beta E(\boldsymbol{v}',\boldsymbol{h}')}}{\sum_{\boldsymbol{v}'',\boldsymbol{h}''} e^{-\beta E(\boldsymbol{v}'',\boldsymbol{h}'')}}\frac{\partial E(\boldsymbol{v}',\boldsymbol{h}')}{\partial \theta_j}\\
&= -\beta \sum_{\boldsymbol{h}} \frac{\frac{1}{Z}e^{-\beta E(\boldsymbol{v},\boldsymbol{h})}}{\frac{1}{Z}\sum_{\boldsymbol{h}'} e^{-\beta E(\boldsymbol{v},\boldsymbol{h}')}}\frac{\partial E(\boldsymbol{v},\boldsymbol{h})}{\partial \theta_j} + \beta \sum_{\boldsymbol{v}',\boldsymbol{h}'} \frac{e^{-\beta E(\boldsymbol{v}',\boldsymbol{h}')}}{Z}\frac{\partial E(\boldsymbol{v}',\boldsymbol{h}')}{\partial \theta_j}\\
&= -\beta \sum_{\boldsymbol{h}} \frac{p(\boldsymbol{v},\boldsymbol{h})}{p(\boldsymbol{v})}\frac{\partial E(\boldsymbol{v},\boldsymbol{h})}{\partial \theta_j} + \beta \sum_{\boldsymbol{v}',\boldsymbol{h}'} p(\boldsymbol{v}',\boldsymbol{h}')\frac{\partial E(\boldsymbol{v}',\boldsymbol{h}')}{\partial \theta_j}\\
&= -\beta \sum_{\boldsymbol{h}} p(\boldsymbol{h}|\boldsymbol{v})\frac{\partial E(\boldsymbol{v},\boldsymbol{h})}{\partial \theta_j} + \beta \sum_{\boldsymbol{v}',\boldsymbol{h}'} p(\boldsymbol{v}',\boldsymbol{h}')\frac{\partial E(\boldsymbol{v}',\boldsymbol{h}')}{\partial \theta_j}\\
&= \beta\left(\mathbb{E}\left(\frac{\partial E}{\partial \theta_j}\right) - \mathbb{E}\left(\frac{\partial E}{\partial \theta_j}\middle|\, \boldsymbol{v}\right)\right)
\end{aligned} \tag{1}$$

The gradient of KL Divergence is estimated as:

$$\begin{aligned}\frac{\partial D_{KL}(q||p)}{\partial\theta_j} &= -\sum_{\boldsymbol{v}\in\{\boldsymbol{v}^1,\ldots,\boldsymbol{v}^D\}} q(\boldsymbol{v})\frac{\partial}{\partial\theta_i}\left(\ln\frac{p(\boldsymbol{v})}{q(\boldsymbol{v})}\right)\\ &= \beta\left(-\mathbb{E}\left(\frac{\partial E}{\partial\theta_j}\right) + \sum_{\boldsymbol{v}\in\{\boldsymbol{v}^1,\ldots,\boldsymbol{v}^D\}} q(\boldsymbol{v})\mathbb{E}\left(\frac{\partial E}{\partial\theta_j}\middle|\,\boldsymbol{v}\right)\right)\end{aligned} \tag{2}$$

B.2 Gradient of Negative Conditional Log-likelihood

$$\begin{aligned}\frac{\partial\mathcal{N}}{\partial\theta_j} &= -\sum_{[\boldsymbol{v}^I,\boldsymbol{v}^O]\in\{\boldsymbol{v}^1,\ldots,\boldsymbol{v}^D\}}\left(\frac{\partial\ln\sum_{\boldsymbol{h''}} e^{-\beta E(\boldsymbol{v}^I,\boldsymbol{v}^O,\boldsymbol{h}')}}{\partial\theta_j} - \frac{\partial\ln\sum_{\boldsymbol{v'}^O,\boldsymbol{h}'} e^{-\beta E(\boldsymbol{v}^I,\boldsymbol{v'}^O,\boldsymbol{h}')}}{\partial\theta_j}\right)\\
&= \beta\sum_{[\boldsymbol{v}^I,\boldsymbol{v}^O]\in\{\boldsymbol{v}^1,\ldots,\boldsymbol{v}^D\}}\left(\sum_{\boldsymbol{h''}}\frac{Zp_\theta(\boldsymbol{v}^I,\boldsymbol{v}^O,\boldsymbol{h''})}{Zp_\theta(\boldsymbol{v}^I,\boldsymbol{v}^O)}\frac{\partial E(\boldsymbol{v}^I,\boldsymbol{v}^O,\boldsymbol{h}'')}{\partial\theta_j} - \sum_{\boldsymbol{v'}^O,\boldsymbol{h}'}\frac{Zp_\theta(\boldsymbol{v}^I,\boldsymbol{v'}^O,\boldsymbol{h}')}{Zp_\theta(\boldsymbol{v}^I)}\frac{\partial E(\boldsymbol{v}^I,\boldsymbol{v'}^O,\boldsymbol{h}')}{\partial\theta_j}\right)\\
&= \beta\sum_{[\boldsymbol{v}^I,\boldsymbol{v}^O]\in\{\boldsymbol{v}^1,\ldots,\boldsymbol{v}^D\}}\left(\sum_{\boldsymbol{h}''} p(\boldsymbol{h}''|\boldsymbol{v}^I,\boldsymbol{v}^O)\frac{\partial E(\boldsymbol{v}^I,\boldsymbol{v}^O,\boldsymbol{h}'')}{\partial\theta_j} - \sum_{\boldsymbol{v'}^O,\boldsymbol{h}'} p(\boldsymbol{v'}^O,\boldsymbol{h}'|\boldsymbol{v}^I)\frac{\partial E(\boldsymbol{v}^I,\boldsymbol{v'}^O,\boldsymbol{h}')}{\partial\theta_j}\right)\\
&= \beta\sum_{[\boldsymbol{v}^I,\boldsymbol{v}^O]\in\{\boldsymbol{v}^1,\ldots,\boldsymbol{v}^D\}}\left(\mathbb{E}\left(\frac{\partial E}{\partial\theta_j}\middle|\,\boldsymbol{v^I},\boldsymbol{v^O}\right) - \mathbb{E}\left(\frac{\partial E}{\partial\theta_j}\middle|\,\boldsymbol{v^I}\right)\right)\end{aligned} \tag{3}$$

B.3 Hessian of KL Divergence

Hessian of Log-likelihood for a single data is estimated first. It uses the fact that in the case of Ising type energy, $\frac{\partial^2 E}{\partial\theta_i\partial\theta_j} = 0$ for all possible i and j.

$$\begin{aligned}\frac{\partial^2\ln p}{\partial\theta_i\partial\theta_j}(\boldsymbol{v}) =& -\beta\sum_{\boldsymbol{h}}\frac{\partial p(\boldsymbol{h}|\boldsymbol{v})}{\partial\theta_i}\frac{\partial E(\boldsymbol{v},\boldsymbol{h})}{\partial\theta_j} + \beta\sum_{\boldsymbol{v}',\boldsymbol{h}'}\frac{\partial p(\boldsymbol{v}',\boldsymbol{h}')}{\partial\theta_i}\frac{\partial E(\boldsymbol{v}',\boldsymbol{h}')}{\partial\theta_j}\\
=& -\beta\sum_{\boldsymbol{h}}\frac{\partial}{\partial\theta_i}\left(\frac{e^{-\beta E(\boldsymbol{v},\boldsymbol{h})}}{\sum_{\boldsymbol{h}''} e^{-\beta E(\boldsymbol{v},\boldsymbol{h}'')}}\right)\frac{\partial E(\boldsymbol{v},\boldsymbol{h})}{\partial\theta_j} + \beta\sum_{\boldsymbol{v}',\boldsymbol{h}'}\frac{\partial}{\partial\theta_i}\left(\frac{e^{-\beta E(\boldsymbol{v}',\boldsymbol{h}')}}{\sum_{\boldsymbol{v}''',\boldsymbol{h}'''} e^{-\beta E(\boldsymbol{v}''',\boldsymbol{h}''')}}\right)\frac{\partial E(\boldsymbol{v}',\boldsymbol{h}')}{\partial\theta_j}\\
=&\beta^2\sum_{\boldsymbol{h}}\left(p(\boldsymbol{h}|\boldsymbol{v})\frac{\partial E(\boldsymbol{v},\boldsymbol{h})}{\partial\theta_i} - p(\boldsymbol{h}|\boldsymbol{v})\sum_{\boldsymbol{h}''} p(\boldsymbol{h}''|\boldsymbol{v})\frac{\partial E(\boldsymbol{v},\boldsymbol{h}'')}{\partial\theta_i}\right)\frac{\partial E(\boldsymbol{v},\boldsymbol{h})}{\partial\theta_j}\\
&-\beta^2\sum_{\boldsymbol{v}',\boldsymbol{h}'}\left(p(\boldsymbol{v}',\boldsymbol{h}')\frac{\partial E(\boldsymbol{v}',\boldsymbol{h}')}{\partial\theta_i} - p(\boldsymbol{v}',\boldsymbol{h}')\sum_{\boldsymbol{v}''',\boldsymbol{h}'''} p(\boldsymbol{v}''',\boldsymbol{h}''')\frac{\partial E(\boldsymbol{v}''',\boldsymbol{h}''')}{\partial\theta_i}\right)\frac{\partial E(\boldsymbol{v}',\boldsymbol{h}')}{\partial\theta_j}\\
=&\beta^2\left(\sum_{\boldsymbol{h}} p(\boldsymbol{h}|\boldsymbol{v})\frac{\partial E(\boldsymbol{v},\boldsymbol{h})}{\partial\theta_i}\frac{\partial E(\boldsymbol{v},\boldsymbol{h})}{\partial\theta_j}\right) - \beta^2\left(\sum_{\boldsymbol{h}} p(\boldsymbol{h}|\boldsymbol{v})\frac{\partial E(\boldsymbol{v},\boldsymbol{h})}{\partial\theta_i}\right)\left(\sum_{\boldsymbol{h}} p(\boldsymbol{h}|\boldsymbol{v})\frac{\partial E(\boldsymbol{v},\boldsymbol{h})}{\partial\theta_j}\right)\\
&-\beta^2\left(\sum_{\boldsymbol{v}',\boldsymbol{h}'} p(\boldsymbol{v}',\boldsymbol{h}')\frac{\partial E(\boldsymbol{v}',\boldsymbol{h}')}{\partial\theta_i}\frac{\partial E(\boldsymbol{v}',\boldsymbol{h}')}{\partial\theta_j}\right) + \beta^2\left(\sum_{\boldsymbol{v}',\boldsymbol{h}'} p(\boldsymbol{v}',\boldsymbol{h}')\frac{\partial E(\boldsymbol{v}',\boldsymbol{h}')}{\partial\theta_i}\right)\left(\sum_{\boldsymbol{v}',\boldsymbol{h}'} p(\boldsymbol{v}',\boldsymbol{h}')\frac{\partial E(\boldsymbol{v}',\boldsymbol{h}')}{\partial\theta_j}\right)\\
=&\beta^2\left(\mathrm{Cov}\left(\frac{\partial E}{\partial\theta_i},\frac{\partial E}{\partial\theta_j}\middle|\,\boldsymbol{v}\right) - \mathrm{Cov}\left(\frac{\partial E}{\partial\theta_i},\frac{\partial E}{\partial\theta_j}\right)\right)\end{aligned} \tag{4}$$

The hessian of KL Divergence is estimated as:

$$\frac{\partial^2 D_{KL}(q||p)}{\partial\theta_i\partial\theta_j} = \beta^2 \left(\mathrm{Cov}\left(\frac{\partial E}{\partial\theta_i}, \frac{\partial E}{\partial\theta_j}\right) - \sum_{\boldsymbol{v}\in\{\boldsymbol{v}^1,\ldots,\boldsymbol{v}^D\}} q(\boldsymbol{v})\, \mathrm{Cov}\left(\frac{\partial E}{\partial\theta_i}, \frac{\partial E}{\partial\theta_j}\middle| \boldsymbol{v}\right)\right) \tag{5}$$

B.4 Hessian of Negative Conditional Log-likelihood for a single data

Hessian for a single data is estimated as: $\boldsymbol{v} \equiv [\boldsymbol{v}^I, \boldsymbol{v}^O]$

$$\begin{aligned}
\frac{\partial^2 \mathcal{N}}{\partial\theta_i\partial\theta_j} =& \beta \left(\sum_{\boldsymbol{h}''} \frac{\partial p(\boldsymbol{h}''|\boldsymbol{v}^I,\boldsymbol{v}^O)}{\partial\theta_i} \frac{\partial E(\boldsymbol{v}^I,\boldsymbol{v}^O,\boldsymbol{h}'')}{\partial\theta_j} - \sum_{\boldsymbol{v'}^O,\boldsymbol{h}'} \frac{\partial p(\boldsymbol{v'}^O,\boldsymbol{h}'|\boldsymbol{v}^I)}{\partial\theta_i} \frac{\partial E(\boldsymbol{v}^I,\boldsymbol{v'}^O,\boldsymbol{h}')}{\partial\theta_j} \right) \\
=& \beta \left(\sum_{\boldsymbol{h}''} \frac{\partial}{\partial\theta_i} \left(\frac{e^{-\beta E(\boldsymbol{v}^I,\boldsymbol{v}^O,\boldsymbol{h}'')}}{\sum_{\overline{\boldsymbol{h}}} e^{-\beta E(\boldsymbol{v}^I,\boldsymbol{v}^O,\overline{\boldsymbol{h}})}} \right) \frac{\partial E(\boldsymbol{v}^I,\boldsymbol{v}^O,\boldsymbol{h}'')}{\partial\theta_j} - \sum_{\boldsymbol{v'}^O,\boldsymbol{h}'} \frac{\partial}{\partial\theta_i} \left(\frac{e^{-\beta E(\boldsymbol{v}^I,\boldsymbol{v'}^O,\boldsymbol{h}')}}{\sum_{\widetilde{\boldsymbol{v}}^O,\widetilde{\boldsymbol{h}}} e^{-\beta E(\boldsymbol{v}^I,\widetilde{\boldsymbol{v}}^O,\widetilde{\boldsymbol{h}})}} \right) \frac{\partial E(\boldsymbol{v}^I,\boldsymbol{v'}^O,\boldsymbol{h}')}{\partial\theta_j} \right) \\
=& -\beta^2 \left(\sum_{\boldsymbol{h}''} \left(p(\boldsymbol{h}''|\boldsymbol{v}^I,\boldsymbol{v}^O) \frac{\partial E(\boldsymbol{v}^I,\boldsymbol{v}^O,\boldsymbol{h}'')}{\partial\theta_i} - \sum_{\overline{\boldsymbol{h}}} p(\overline{\boldsymbol{h}}|\boldsymbol{v}^I,\boldsymbol{v}^O) \frac{\partial E(\boldsymbol{v}^I,\boldsymbol{v}^O,\overline{\boldsymbol{h}})}{\partial\theta_i} \right) \frac{\partial E(\boldsymbol{v}^I,\boldsymbol{v}^O,\boldsymbol{h}'')}{\partial\theta_j} \right. \\
& \left. - \sum_{\boldsymbol{v'}^O,\boldsymbol{h}'} \left(p(\boldsymbol{v'}^O,\boldsymbol{h}'|\boldsymbol{v}^I) \frac{\partial E(\boldsymbol{v}^I,\boldsymbol{v'}^O,\boldsymbol{h}')}{\partial\theta_i} - \sum_{\widetilde{\boldsymbol{v}}^O\widetilde{\boldsymbol{h}}} p(\widetilde{\boldsymbol{v}}^O,\widetilde{\boldsymbol{h}}|\boldsymbol{v}^I) \frac{\partial E(\boldsymbol{v}^I,\widetilde{\boldsymbol{v}}^O,\widetilde{\boldsymbol{h}})}{\partial\theta_i} \right) \frac{\partial E(\boldsymbol{v}^I,\boldsymbol{v'}^O,\boldsymbol{h}')}{\partial\theta_j} \right) \\
=& -\beta^2 \left(\left(\sum_{\boldsymbol{h}''} p(\boldsymbol{h}''|\boldsymbol{v}^I,\boldsymbol{v}^O) \frac{\partial E(\boldsymbol{v}^I,\boldsymbol{v}^O,\boldsymbol{h}'')}{\partial\theta_i} \frac{\partial E(\boldsymbol{v}^I,\boldsymbol{v}^O,\boldsymbol{h}'')}{\partial\theta_j} \right) \right. \\
& - \left(\sum_{\overline{\boldsymbol{h}}} p(\overline{\boldsymbol{h}}|\boldsymbol{v}^I,\boldsymbol{v}^O) \frac{\partial E(\boldsymbol{v}^I,\boldsymbol{v}^O,\overline{\boldsymbol{h}})}{\partial\theta_i} \right) \left(\sum_{\boldsymbol{h}''} p(\boldsymbol{h}''|\boldsymbol{v}^I,\boldsymbol{v}^O) \frac{\partial E(\boldsymbol{v}^I,\boldsymbol{v}^O,\boldsymbol{h}'')}{\partial\theta_j} \right) \\
& - \left(\sum_{\boldsymbol{v'}^O,\boldsymbol{h}'} p(\boldsymbol{v'}^O,\boldsymbol{h}'|\boldsymbol{v}^I) \frac{\partial E(\boldsymbol{v}^I,\boldsymbol{v'}^O,\boldsymbol{h}')}{\partial\theta_i} \frac{\partial E(\boldsymbol{v}^I,\boldsymbol{v'}^O,\boldsymbol{h}')}{\partial\theta_j} \right) \\
& \left. + \left(\sum_{\widetilde{\boldsymbol{v}}^O\widetilde{\boldsymbol{h}}} p(\widetilde{\boldsymbol{v}}^O,\widetilde{\boldsymbol{h}}|\boldsymbol{v}^I) \frac{\partial E(\boldsymbol{v}^I,\widetilde{\boldsymbol{v}}^O,\widetilde{\boldsymbol{h}})}{\partial\theta_i} \right) \left(\sum_{\boldsymbol{v'}^O,\boldsymbol{h}'} p(\boldsymbol{v'}^O,\boldsymbol{h}'|\boldsymbol{v}^I) \frac{\partial E(\boldsymbol{v}^I,\boldsymbol{v'}^O,\boldsymbol{h}')}{\partial\theta_j} \right) \right) \\
=& \beta^2 \left(\mathrm{Cov}\left(\frac{\partial E}{\partial\theta_i}, \frac{\partial E}{\partial\theta_j}\middle| \boldsymbol{v}^I\right) - \mathrm{Cov}\left(\frac{\partial E}{\partial\theta_i}, \frac{\partial E}{\partial\theta_j}\middle| \boldsymbol{v}\right) \right)
\end{aligned} \tag{6}$$

Now considering the visible data, $[\boldsymbol{v}^I, \boldsymbol{v}^O] \in \{\boldsymbol{v}^1, \ldots, \boldsymbol{v}^D\}$,

$$\frac{\partial^2 \mathcal{N}}{\partial\theta_i\partial\theta_j} = \beta^2 \sum_{[\boldsymbol{v}^I,\boldsymbol{v}^O]\in\{\boldsymbol{v}^1,\ldots,\boldsymbol{v}^D\}} \left(\mathrm{Cov}\left(\frac{\partial E}{\partial\theta_i}, \frac{\partial E}{\partial\theta_j}\middle| \boldsymbol{v}^I\right) - \mathrm{Cov}\left(\frac{\partial E}{\partial\theta_i}, \frac{\partial E}{\partial\theta_j}\middle| \boldsymbol{v}^I, \boldsymbol{v}^O\right) \right) \tag{7}$$

B.5 Derivative of KL Divergence w.r.t. Inverse temperature

$$\frac{dD_{KL}}{d\beta} = -\sum_{\boldsymbol{v}\in\{\boldsymbol{v}^1,\ldots,\boldsymbol{v}^D\}} q(\boldsymbol{v}) \frac{d}{d\beta} \ln \frac{p(\boldsymbol{v})}{q(\boldsymbol{v})} = -\sum_{\boldsymbol{v}\in\{\boldsymbol{v}^1,\ldots,\boldsymbol{v}^D\}} \frac{q(\boldsymbol{v})}{p(\boldsymbol{v})} \frac{d}{d\beta} \frac{\sum_{\boldsymbol{h}} e^{-\beta E(\boldsymbol{v},\boldsymbol{h})}}{\sum_{\boldsymbol{v}',\boldsymbol{h}'} e^{-\beta E(\boldsymbol{v}',\boldsymbol{h}')}}$$

$$= -\sum_{\boldsymbol{v}\in\{\boldsymbol{v}^1,\ldots,\boldsymbol{v}^D\}} \frac{q(\boldsymbol{v})}{p(\boldsymbol{v})} \left(\frac{-\sum_{\boldsymbol{h}} E(\boldsymbol{v},\boldsymbol{h}) e^{-\beta E(\boldsymbol{v},\boldsymbol{h})}}{\sum_{\boldsymbol{v}',\boldsymbol{h}'} e^{-\beta E(\boldsymbol{v}',\boldsymbol{h}')}} + \frac{\left(\sum_{\boldsymbol{h}} e^{-\beta E(\boldsymbol{v},\boldsymbol{h})}\right)\left(\sum_{\boldsymbol{v}',\boldsymbol{h}'} E(\boldsymbol{v}',\boldsymbol{h}') e^{-\beta E(\boldsymbol{v}',\boldsymbol{h}')}\right)}{\left(\sum_{\boldsymbol{v}'',\boldsymbol{h}''} e^{-\beta E(\boldsymbol{v}'',\boldsymbol{h}'')}\right)^2} \right)$$

$$= \sum_{\boldsymbol{v}\in\{\boldsymbol{v}^1,\ldots,\boldsymbol{v}^D\}} \frac{q(\boldsymbol{v})}{p(\boldsymbol{v})} \left(\sum_{\boldsymbol{h}} E(\boldsymbol{v},\boldsymbol{h}) p(\boldsymbol{v},\boldsymbol{h}) - p(\boldsymbol{v}) \sum_{\boldsymbol{v}',\boldsymbol{h}'} E(\boldsymbol{v}',\boldsymbol{h}') p(\boldsymbol{v}',\boldsymbol{h}') \right)$$

$$= \sum_{\boldsymbol{v}\in\{\boldsymbol{v}^1,\ldots,\boldsymbol{v}^D\}} q(\boldsymbol{v}) \left(\sum_{\boldsymbol{h}} E(\boldsymbol{v},\boldsymbol{h}) p(\boldsymbol{h}|\boldsymbol{v}) - \sum_{\boldsymbol{v}',\boldsymbol{h}'} E(\boldsymbol{v}',\boldsymbol{h}') p(\boldsymbol{v}',\boldsymbol{h}') \right)$$

$$= -\mathbb{E}_{\boldsymbol{v},\boldsymbol{h}}(E) + \sum_{\boldsymbol{v}\in\{\boldsymbol{v}^1,\ldots,\boldsymbol{v}^D\}} q(\boldsymbol{v}) \sum_{\boldsymbol{h}} E(\boldsymbol{v},\boldsymbol{h}) p(\boldsymbol{h}|\boldsymbol{v})$$

$$\frac{d^2 D_{KL}}{d\beta^2} = \sum_{\boldsymbol{v}\in\{\boldsymbol{v}^1,\ldots,\boldsymbol{v}^D\}} q(\boldsymbol{v}) \left(\sum_{\boldsymbol{h}} E(\boldsymbol{v},\boldsymbol{h}) \frac{d}{d\beta} p(\boldsymbol{h}|\boldsymbol{v}) - \sum_{\boldsymbol{v}',\boldsymbol{h}'} E(\boldsymbol{v}',\boldsymbol{h}') \frac{d}{d\beta} p(\boldsymbol{v}',\boldsymbol{h}') \right)$$

$$= \sum_{\boldsymbol{v}\in\{\boldsymbol{v}^1,\ldots,\boldsymbol{v}^D\}} q(\boldsymbol{v}) \left(\sum_{\boldsymbol{h}} E(\boldsymbol{v},\boldsymbol{h}) \underbrace{\frac{d}{d\beta} \frac{e^{-\beta E(\boldsymbol{v},\boldsymbol{h})}}{\sum_{\boldsymbol{h}''} e^{-\beta E(\boldsymbol{v},\boldsymbol{h}'')}}}_{\text{Term } I} - \sum_{\boldsymbol{v}',\boldsymbol{h}'} E(\boldsymbol{v}',\boldsymbol{h}') \underbrace{\frac{d}{d\beta} \frac{e^{-\beta E(\boldsymbol{v}',\boldsymbol{h}')}}{\sum_{\boldsymbol{v}'',\boldsymbol{h}''} e^{-\beta E(\boldsymbol{v}'',\boldsymbol{h}'')}}}_{\text{Term } II} \right)$$

Term I is evaluated as:

$$\frac{d}{d\beta} \frac{e^{-\beta E(\boldsymbol{v},\boldsymbol{h})}}{\sum_{\boldsymbol{h}''} e^{-\beta E(\boldsymbol{v},\boldsymbol{h}'')}} = -\frac{E(\boldsymbol{v},\boldsymbol{h}) e^{-\beta E(\boldsymbol{v},\boldsymbol{h})}}{\sum_{\boldsymbol{h}''} e^{-\beta E(\boldsymbol{v},\boldsymbol{h}'')}} + \frac{e^{-\beta E(\boldsymbol{v},\boldsymbol{h})} \sum_{\boldsymbol{h}'} E(\boldsymbol{v},\boldsymbol{h}') e^{-\beta E(\boldsymbol{v},\boldsymbol{h}')}}{\left(\sum_{\boldsymbol{h}''} e^{-\beta E(\boldsymbol{v},\boldsymbol{h}'')}\right)^2}$$

$$= -E(\boldsymbol{v},\boldsymbol{h}) p(\boldsymbol{h}|\boldsymbol{v}) + p(\boldsymbol{h}|\boldsymbol{v}) \sum_{\boldsymbol{h}'} E(\boldsymbol{v},\boldsymbol{h}') p(\boldsymbol{h}'|\boldsymbol{v})$$

Term II is evaluated as:

$$\frac{d}{d\beta} \frac{e^{-\beta E(\boldsymbol{v}',\boldsymbol{h}')}}{\sum_{\boldsymbol{v}'',\boldsymbol{h}''} e^{-\beta E(\boldsymbol{v}'',\boldsymbol{h}'')}} = -\frac{E(\boldsymbol{v}',\boldsymbol{h}') e^{-\beta E(\boldsymbol{v}',\boldsymbol{h}')}}{Z} + \frac{e^{-\beta E(\boldsymbol{v}',\boldsymbol{h}')} \sum_{\boldsymbol{v}'',\boldsymbol{h}''} E(\boldsymbol{v}'',\boldsymbol{h}'') e^{-\beta E(\boldsymbol{v}'',\boldsymbol{h}'')}}{Z^2}$$

$$= -E(\boldsymbol{v}',\boldsymbol{h}') p(\boldsymbol{v}',\boldsymbol{h}') + p(\boldsymbol{v}',\boldsymbol{h}') \sum_{\boldsymbol{v}'',\boldsymbol{h}''} E(\boldsymbol{v}'',\boldsymbol{h}'') p(\boldsymbol{v}'',\boldsymbol{h}'')$$

Combining the two terms:

$$\frac{d^2 D_{KL}}{d\beta^2} = \sum_{\boldsymbol{v}\in\{\boldsymbol{v}^1,\ldots,\boldsymbol{v}^D\}} q(\boldsymbol{v}) \left(\sum_{\boldsymbol{h}} -E^2(\boldsymbol{v},\boldsymbol{h}) p(\boldsymbol{h}|\boldsymbol{v}) + \left(\sum_{\boldsymbol{h}'} E(\boldsymbol{v},\boldsymbol{h}') p(\boldsymbol{h}'|\boldsymbol{v}) \right)^2 \right.$$

$$\left. + \sum_{\boldsymbol{v}',\boldsymbol{h}'} E^2(\boldsymbol{v}',\boldsymbol{h}') p(\boldsymbol{v}',\boldsymbol{h}') - \left(\sum_{\boldsymbol{v}'',\boldsymbol{h}''} E(\boldsymbol{v}'',\boldsymbol{h}'') p(\boldsymbol{v}'',\boldsymbol{h}'') \right)^2 \right)$$

$$= \sum_{\boldsymbol{v}\in\{\boldsymbol{v}^1,\ldots,\boldsymbol{v}^D\}} q(\boldsymbol{v}) \left(-\operatorname{Var}(E|\boldsymbol{v}) + \operatorname{Var}(E) \right)$$

B.6 Derivative of Negative Like-Likelihood w.r.t. Inverse temperature

The derivative of log-likelihood of conditional probability for a single data, $\boldsymbol{v} \equiv [\boldsymbol{v}^I, \boldsymbol{v}^O]$ is calculated first:

$$
\begin{aligned}
\frac{d \ln p(\boldsymbol{v}|\boldsymbol{v}^I)}{d\beta} &= \frac{d}{d\beta}\left(\ln \frac{p(\boldsymbol{v})}{p(\boldsymbol{v}^I)}\right) = \frac{d}{d\beta}\left(\ln \sum_{\boldsymbol{h}} e^{-\beta E(\boldsymbol{v},\boldsymbol{h})} - \ln \sum_{\overline{\boldsymbol{v}}^O,\boldsymbol{h}} e^{-\beta E(\boldsymbol{v}^I,\overline{\boldsymbol{v}}^O,\boldsymbol{h})}\right) \\
&= \sum_{\boldsymbol{h}} -E(\boldsymbol{v},\boldsymbol{h}) \frac{e^{-\beta E(\boldsymbol{v},\boldsymbol{h})}}{\sum_{\boldsymbol{h}'} e^{-\beta E(\boldsymbol{v},\boldsymbol{h}')}} + \sum_{\boldsymbol{v}^{O'},\boldsymbol{h}'} E(\boldsymbol{v}^I,\boldsymbol{v}^{O'},\boldsymbol{h}') \frac{e^{-\beta E(\boldsymbol{v}^I,\boldsymbol{v}^{O'},\boldsymbol{h}')}}{\sum_{\boldsymbol{v}^{O''},\boldsymbol{h}''} e^{-\beta E(\boldsymbol{v}^I,\boldsymbol{v}^{O''},\boldsymbol{h}'')}} \\
&= \sum_{\boldsymbol{h}} -E(\boldsymbol{v},\boldsymbol{h}) p(\boldsymbol{v},\boldsymbol{h}|\boldsymbol{v}) + \sum_{\boldsymbol{v}^{O'},\boldsymbol{h}'} E(\boldsymbol{v}^I,\boldsymbol{v}^{O'},\boldsymbol{h}') p(\boldsymbol{v}^I,\boldsymbol{v}^{O'},\boldsymbol{h}|\boldsymbol{v}^I) \\
&= -\mathbb{E}(E|\boldsymbol{v}) + \mathbb{E}(E|\boldsymbol{v}^I)
\end{aligned}
$$

The second derivative is estimated as

$$
\frac{d^2 \ln p(\boldsymbol{v}|\boldsymbol{v}^I)}{d\beta^2} = \sum_{\boldsymbol{h}} E(\boldsymbol{v},\boldsymbol{h}) \underbrace{\frac{d}{d\beta} \frac{e^{-\beta E(\boldsymbol{v},\boldsymbol{h})}}{\sum_{\boldsymbol{h}'} e^{-\beta E(\boldsymbol{v},\boldsymbol{h}')}}}_{\text{Term } I} - \sum_{\boldsymbol{v}^{O'},\boldsymbol{h}'} E(\boldsymbol{v}^I,\boldsymbol{v}^{O'},\boldsymbol{h}') \underbrace{\frac{d}{d\beta} \frac{e^{-\beta E(\boldsymbol{v}^I,\boldsymbol{v}^{O'},\boldsymbol{h}')}}{\sum_{\boldsymbol{v}^{O''},\boldsymbol{h}''} e^{-\beta E(\boldsymbol{v}^I,\boldsymbol{v}^{O''},\boldsymbol{h}'')}}}_{\text{Term } II}
$$

Term I is evaluated as:

$$
\begin{aligned}
\frac{d}{d\beta} \frac{e^{-\beta E(\boldsymbol{v},\boldsymbol{h})}}{\sum_{\boldsymbol{h}'} e^{-\beta E(\boldsymbol{v},\boldsymbol{h}')}} &= -\frac{E(\boldsymbol{v},\boldsymbol{h}) e^{-\beta E(\boldsymbol{v},\boldsymbol{h})}}{\sum_{\boldsymbol{h}'} e^{-\beta E(\boldsymbol{v},\boldsymbol{h}')}} + \frac{e^{-\beta E(\boldsymbol{v},\boldsymbol{h})} \sum_{\boldsymbol{h}'} E(\boldsymbol{v},\boldsymbol{h}') e^{-\beta E(\boldsymbol{v},\boldsymbol{h}')}}{\left(\sum_{\boldsymbol{h}''} e^{-\beta E(\boldsymbol{v},\boldsymbol{h}'')}\right)^2} \\
&= -E(\boldsymbol{v},\boldsymbol{h}) p(\boldsymbol{h}|\boldsymbol{v}) + p(\boldsymbol{h}|\boldsymbol{v}) \sum_{\boldsymbol{h}'} E(\boldsymbol{v},\boldsymbol{h}') p(\boldsymbol{h}'|\boldsymbol{v}) \\
&= -E(\boldsymbol{v},\boldsymbol{h}) p(\boldsymbol{h}|\boldsymbol{v}) + p(\boldsymbol{h}|\boldsymbol{v}) \mathbb{E}(E|\boldsymbol{v})
\end{aligned}
$$

Term II is evaluated as:

$$
\begin{aligned}
\frac{d}{d\beta} \frac{e^{-\beta E(\boldsymbol{v}^I,\boldsymbol{v}^{O'},\boldsymbol{h}')}}{\sum_{\boldsymbol{v}^{O''},\boldsymbol{h}''} e^{-\beta E(\boldsymbol{v}^I,\boldsymbol{v}^{O''},\boldsymbol{h}'')}} &= -E(\boldsymbol{v}^I,\boldsymbol{v}^{O'},\boldsymbol{h}') \frac{e^{-\beta E(\boldsymbol{v}^I,\boldsymbol{v}^{O'},\boldsymbol{h}')}}{\sum_{\boldsymbol{v}^{O''},\boldsymbol{h}''} e^{-\beta E(\boldsymbol{v}^I,\boldsymbol{v}^{O''},\boldsymbol{h}'')}} + \\
&\quad \frac{e^{-\beta E(\boldsymbol{v}^I,\boldsymbol{v}^{O'},\boldsymbol{h}')} \sum_{\boldsymbol{v}^{O''},\boldsymbol{h}''} E(\boldsymbol{v}^I,\boldsymbol{v}^{O''},\boldsymbol{h}'') e^{-\beta E(\boldsymbol{v}^I,\boldsymbol{v}^{O''},\boldsymbol{h}'')}}{\left(\sum_{\boldsymbol{v}^{O'''},\boldsymbol{h}'''} e^{-\beta E(\boldsymbol{v}^I,\boldsymbol{v}^{O'''},\boldsymbol{h}''')}\right)^2} \\
&= -E(\boldsymbol{v}^I,\boldsymbol{v}^{O'},\boldsymbol{h}') p(\boldsymbol{v}^{O'},\boldsymbol{h}'|\boldsymbol{v}^I) + p(\boldsymbol{v}^{O'},\boldsymbol{h}'|\boldsymbol{v}^I) \mathbb{E}(E|\boldsymbol{v}^I)
\end{aligned}
$$

Combining the two terms:

$$
\begin{aligned}
\frac{d^2 \ln p(\boldsymbol{v}|\boldsymbol{v}^I)}{d\beta^2} &= \sum_{\boldsymbol{h}} -E^2(\boldsymbol{v},\boldsymbol{h}) p(\boldsymbol{h}|\boldsymbol{v}) + \mathbb{E}(E|\boldsymbol{v}) \sum_{\boldsymbol{h}} E(\boldsymbol{v},\boldsymbol{h}) p(\boldsymbol{h}|\boldsymbol{v}) \\
&\quad + \sum_{\boldsymbol{v}^{O'},\boldsymbol{h}'} E^2(\boldsymbol{v}^I,\boldsymbol{v}^{O'},\boldsymbol{h}') p(\boldsymbol{v}^{O'},\boldsymbol{h}'|\boldsymbol{v}^I) - \mathbb{E}(E|\boldsymbol{v}^I) \sum_{\boldsymbol{v}^{O'},\boldsymbol{h}'} E(\boldsymbol{v}^I,\boldsymbol{v}^{O'},\boldsymbol{h}') p(\boldsymbol{v}^{O'},\boldsymbol{h}'|\boldsymbol{v}^I)
\end{aligned}
$$

$$= -\mathbb{E}(E^2|\boldsymbol{v}) + \mathbb{E}^2(E|\boldsymbol{v}) + \mathbb{E}(E^2|\boldsymbol{v}^I) - \mathbb{E}^2(E|\boldsymbol{v}^I)$$
$$= -\operatorname{Var}(E|\boldsymbol{v}) + \operatorname{Var}(E|\boldsymbol{v}^I)$$

The first derivative of Negative conditional log-likelihood is estimated as:

$$\frac{d\mathcal{N}}{d\beta} = \sum_{[\boldsymbol{v}^I,\boldsymbol{v}^O]\in\{\boldsymbol{v}^1,\ldots,\boldsymbol{v}^D\}} \mathbb{E}(E|\boldsymbol{v}) - \mathbb{E}(E|\boldsymbol{v}^I)$$

The second derivative of Negative conditional log-likelihood is estimated as:

$$\frac{d^2\mathcal{N}}{d\beta^2} = \sum_{[\boldsymbol{v}^I,\boldsymbol{v}^O]\in\{\boldsymbol{v}^1,\ldots,\boldsymbol{v}^D\}} -\operatorname{Var}(E|\boldsymbol{v}) + \operatorname{Var}(E|\boldsymbol{v}^I)$$

Appendix C Change of basis

It is a common practice to define the Ising states as either $\{0, 1\}$ or $\{-1, +1\}$ states. The latter format is employed on the DWave machine. Here, the details about conversion between these formats are presented. For the purpose of discussion, the $\{0, 1\}$ Ising model is represented with variables, $\{\boldsymbol{S}, \{H_i\}_{i=1}^{N_V}, \{J_i\}_{i=1}^{N_C}\}$ and the $\{-1, 1\}$ Ising with model is represented with overlined variables, $\{\overline{\boldsymbol{S}}, \{\overline{H}_i\}_{i=1}^{N_V}, \{\overline{J}_i\}_{i=1}^{N_C}\}$. The three variables represent the state, field energy and interaction energy respectively. The states of the system can be interchanged using the following equation:

$$\overline{\boldsymbol{S}} = 2\boldsymbol{S} - 1 \tag{8}$$

The interaction parameters can be interchanged as:

$$\overline{J}_k = \frac{1}{4}J_k \tag{9}$$

And the field parameter as:

$$\overline{H}_i = \frac{1}{2}H_i + \frac{1}{4}\sum_{\pi(k,1)=i \text{ OR } \pi(k,2)=i} J_k \tag{10}$$

This transformation shifts the energy of each state with a constant value and hence leaves the Boltzmann probability unchanged as required.
